# Exploring the Interplay of Competition and Justice: A Moderated Mediation Model of Competitive Psychological Climate, Workplace Envy, Interpersonal Citizenship Behavior, and Organizational Justice

**DOI:** 10.3390/bs14010005

**Published:** 2023-12-21

**Authors:** Sevcan Yıldız, Engin Üngüren, Ömer Akgün Tekin, Engin Derman

**Affiliations:** 1 Department of Tourism and Travel Services, Social Sciences Vocational School, Akdeniz University, Antalya 07600, Turkey; sevcanyildiz@akdeniz.edu.tr; 2 Department of Business Management, Faculty of Economics, Administrative and Social Sciences, Alanya Alaaddin Keykubat University, Antalya 07450, Turkey; engin.unguren@alanya.edu.tr; 3 Department of Gastronomy and Culinary Arts, Manavgat Faculty of Tourism, Akdeniz University, Antalya 07600, Turkey; 4 Department of Tourism Guidance, Manavgat Faculty of Tourism, Akdeniz University, Antalya 07600, Turkey

**Keywords:** negative acts, competitive psychological climate, workplace envy, interpersonal citizenship behavior, organizational justice

## Abstract

The competitive psychological environment that arises within an organization is widely recognized as a crucial factor impacting employee performance and, indirectly, overall business productivity. Nonetheless, mishandling this environment can result in unforeseen challenges. Thus, a moderated mediation model was employed in this study to ascertain the adverse effects of competitive psychological climate and how to mitigate said effects. Data were collected via a survey of 523 employees of four- and five-star accommodation establishments in Alanya and Manavgat using convenience sampling. This study revealed that a competitive work environment leads to increased workplace envy, which adversely affects interpersonal citizenship behavior. Additionally, it was discovered that workplace envy mediates the effects of competitive climate on interpersonal citizenship behavior. The negative impact of competitive psychological climate on workplace envy and interpersonal citizenship behavior is mitigated by organizational justice. This study’s results offer significant contributions to both theoretical and practical understandings of the potential effects of competitive psychological climate and how to handle them.

## 1. Introduction

Competitive advantage has become a crucial strategic component [1] for tourism enterprises facing the current market environment, where products and services are increasingly homogenized [2]. Hotels compete with their rivals in multiple areas, such as maintaining high service quality standards, satisfying guests’ expectations, implementing appropriate pricing strategies, achieving technological superiority, and enhancing efficiency [3,4]. To gain competitive advantage in all these areas, employees must enhance their performance within the milieu of these competitive factors. Competitive conditions demand that employees participate in enterprise processes by taking on tasks that exceed their job responsibilities [5]. Therefore, creating competitive psychological climate within an organization is believed to enhance employee performance in competitive environments. This environment motivates employees to achieve their goals and contributes positively to the competitiveness of the business [6,7]. Employee performance is a significant factor in the competitive landscape of businesses [8]. In competitive psychological climates, employees engage in a competitive performance [9]. The resulting performance outcomes have an impact on the organization’s competitive abilities [10].

Competitive psychological climate refers to the extent to which employees perceive that their rewards from their organization depend on how their performance compares to that of their peers. This environment encourages employees to concentrate on the performance standards and rewards instituted by the organization, resulting in them comparing their performance with their peers. Consequently, they endeavor to achieve their objectives [11]. An enhanced employee performance due to competitive psychological climate leads to better competitive capabilities for the organization [12]. In such a climate, employees believe that they can obtain organizational rewards through comparing themselves to their colleagues [11,13]. Accordingly, in competitive psychological climate, employees may exhibit superior performance compared to their rivals [14]. Previous research indicates that competitive psychological climate, under certain circumstances, enhances employee performance [15], promotes successful career advancement [16], fosters employee engagement [17], and boosts productivity and innovation within organizations [18]. However, there are also studies that present noteworthy findings about the unfavorable impacts of competitive psychological climate. In multiple studies, competitive psychological climate has been found to generate a negative atmosphere among employees, encourage employees to undermine each other’s work, decrease workplace cooperation [19], amplify stress and uncertainty within the organization [20], and lead to counterproductive workplace conduct [21]. It is understood that competitive psychological climate, initially seen as an attractive factor for business productivity, may, in fact, produce unintended negative consequences. This is due to the inherent nature of such climate, which leads employees to continuously compare their performance with coworkers. On the one hand, this situation encourages employees to make personal and professional investments to enhance their skills, but on the other hand, it has the potential to harm cooperation among colleagues [22,23]. Additionally, competitive psychological climate can lead to disagreements and conflicts among employees [24] while also triggering envy within the workplace [25].

Workplace envy arises when employees feel inferior due to comparisons [26]. Over time, it can hinder collaboration and decrease the productivity of the organization [27]. Although envy, which is widely observed in organizations [28,29], can be a powerful emotion experienced by most individuals throughout their lives, it can also have specific destructive effects [30]. Envy can lead to increased productivity and efficiency in some cases [31]. However, it can also result in negative outcomes such as counterproductive work behavior, social undermining, reduced organizational engagement, and increased turnover intention [32,33].

Envy is defined as a “pattern of thoughts, emotions, and behaviors resulting from an employee’s loss of self-esteem in response to another’s attainment of desired outcomes” [34]. There are two types of envy: benign envy, which is a positive emotional state, and malicious envy, which is considered destructive [35,36]. The primary motivation of an envious individual is to diminish the disparity between themselves and the person they envy by creating equal positions for themselves and the envied individual [37]. Consequently, people can attempt to surpass their inadequacies in order to achieve personal growth, which can be considered a positive action, referred to as benign envy [38]. However, certain employees may choose to withhold personal growth and instead anticipate the regression of those they feel envious of or engage in destructive behavior to prompt their downfall. This kind of envy falls under the category of malicious envy [39]. While envy may prove beneficial for businesses, it can quickly turn into counterproductive work behavior [21,33]. Therefore, it is clear that competitive psychological climate, viewed as a beneficial element in enhancing employee performance and productivity in organizations, may also trigger malicious envy. As such, this study highlights its relevance as a vital research issue by investigating the impact of competitive psychological climate on workplace envy among hotel industry workers. A handful of studies in the literature [22,32,40,41,42] confirm the positive correlation between competitive psychological climate and workplace envy. Thus, we propose the first hypothesis of this study below.

**H1:**  *A competitive psychological climate has a positive impact on workplace envy.*

In this study, workplace envy was examined as a negative outcome of competitive psychological climate. The detrimental effects of workplace envy were also explored, since workplace envy can potentially become a socially destructive force for organizations, leading to organizational dysfunctions and critical system damage [43], causing employees to feel devalued [32], weakening interpersonal relationships, reducing cooperation [44,45], and negatively affecting organizational citizenship behavior [25,46]. Interpersonal citizenship behavior is among the particular concepts addressed within the scope of organizational citizenship [47]. Interpersonal citizenship behavior involves employees assisting each other beyond their job duties to improve individual job performance and strengthen organizational functioning. This behavior can be categorized into two dimensions: task-focused and person-focused interpersonal citizenship behavior [48]. Person-focused interpersonal citizenship behavior pertains to more personal issues. Person-focused interpersonal citizenship behavior is generally associated with the emotional states, self-esteem, and personal qualities of employees. In simpler terms, when employees assist one another with matters related to their emotional well-being, self-esteem, and personal qualities, it falls under the category of person-focused interpersonal citizenship behavior [49]. For instance, employees being available to one another, actively listening, displaying interest, and providing social assistance when needed may fall under the scope of person-focused interpersonal citizenship behavior [47].

Task-focused interpersonal citizenship behavior focuses on tasks and organizational issues rather than personal matters. Instances of task-focused interpersonal citizenship behavior involve workers offering guidance on work-related matters, suggesting novel approaches to work-related problems, aiding in the resolution of work-related problems, and directly helping promote productivity [47,50]. Task-focused interpersonal citizenship behavior is a work-related process that is influenced by the quality of relationships between employees [47]. Strong employee relationships decrease the likelihood of employees being bystanders to each other’s difficulties and promote a cooperative environment [51]. Good employee relations can create a trusting atmosphere within the organization, convincing employees that their interests will be protected when necessary and they will receive solidarity from colleagues [50]. Several studies [48,52,53] have shown positive links between good relations within the organization and interpersonal citizenship behavior. However, just as good relations between employees have a positive effect on interpersonal citizenship behavior, in the opposite case, that is, if the relations within the organization deteriorate, this negativity may reduce interpersonal citizenship behavior. In this context, workplace envy may harm cooperation and collaboration by eroding interpersonal relationships within the organization [44,45]. Additionally, it is closely associated with counterproductive workplace behavior [19] and is considered a factor that reduces interpersonal citizenship behavior [54]. Drawing from these evaluations, we propose the second hypothesis of this study below.

**H2:**  *Workplace envy has a negative impact on interpersonal citizenship behavior.*

In this study, workplace envy is considered a variable negatively impacted by competitive psychological climate, which also negatively affects interpersonal citizenship behavior. Competitive psychological climates promote more social comparisons within an organization, creating an environment conducive to developing workplace envy [55]. In essence, competitive psychological climate is expected to have an adverse impact on workplace envy [25]. Kim et al. [44] found in their study of frontline workers in the hospitality industry that employees compare their performance results with others, leading to workplace envy. This phenomenon is also present among food and beverage department employees, resulting in competitive psychological climate that fosters workplace envy. Workplace envy, resulting from competitive psychological climate, negatively affects interpersonal citizenship behavior by weakening employee relationships [56]. Put differently, employees are more likely to relinquish citizenship behaviors and less likely to express positive sentiments toward the organization or exceed expectations when they covet what others possess [57]. It is hypothesized that envy in the workplace mediates the connection between competitive psychological climate and interpersonal citizenship behavior. Therefore, this study’s third hypothesis is as follows:

**H3:**  *Workplace envy mediates the effect of competitive psychological climate on interpersonal citizenship behavior.*

Another aim of this study is to determine whether organizational justice plays a moderating role in the relationship between competitive psychological climate and workplace envy. Organizational justice is employees’ perception that the decision-making processes, practices, and treatment of employees within the organization are fair [58,59,60]. Existing studies have shown that the concept of organizational justice has dimensions of distributive, procedural, interactional, interpersonal, and information justice [61,62,63]. This study focuses on “distributive justice”, which Colquitt [64] defines as justice regarding the distribution of resources in an organization. In general, organizational justice is a particularly important concept for both the organization and the employees. When employees believe that they are treated fairly in the organization they are in, it contributes to the development of a positive climate in the organization [65]. Employees with similar mental and physical abilities naturally compare the benefits they have received from the organization they work for with those received by their colleagues. Significant differences that emerge during this comparison give rise to envy [66,67]. In fact, when employees develop a sense of envy, it may create a favorable ground for the formation of some negative perceptions about justice [68]. However, the fair distribution of organizational benefits to employees in similar circumstances can be considered a solution to buffer the problem of workplace envy, which is a consequence of competitive psychological climate. On the other hand, organizational justice can also be considered a factor that helps employees in competitive workplace conditions [69,70]. In this context, it can be said that organizational justice plays a key role in competitive psychological climate conditions. In fact, while the provision of organizational justice can contribute to combating the challenges of competitive psychological climate, such as workplace envy [71], the lack of justice can lead to the strengthening of these challenges. This is because employees would perceive competition in a fair organization as less risky for them. Thus, employees will tend not to engage in negative behaviors even under challenging conditions [72]. Within the framework of this information, the fourth and fifth hypotheses of the study are proposed as follows:

**H4:**  *Organizational justice has a moderating role in the relationship between competitive psychological climate and workplace envy.*

**H5:**  *The mediating role of workplace envy in the relationship between competitive psychological climate and citizenship behavior differs according to employees’ perceptions of distributive justice.*

This study is expected to contribute to the literature in several ways. First, this study provides researchers and industry managers with the possible effects of competitive psychological climate—which is designed to improve employee performance and organizational efficiency—on workplace envy and interpersonal citizenship behavior. Second, this research study not only approaches the issue of workplace envy as an outcome, but also tries to reveal the effect of this phenomenon on interpersonal citizenship behavior. Third, as another important point, this study presents findings on the moderating role of organizational justice in the relationship between competitive psychological climate and workplace envy. In this context, findings on the effect of organizational justice on the relationship between competitive psychological climate, workplace envy, and interpersonal citizenship variables are presented. Finally, it is hoped that this study will contribute to social comparison theory (SCT), especially within the framework of the competitive psychological climate factor, which is the main variable of this analysis. This study is hoped to contribute to the theoretical field and applications in the tourism sector by presenting some original results, especially in terms of the preferred model and the selected sample.

Social comparison theory provides the theoretical background for the research. Social comparison can be defined as “the process of thinking about information about one or more people in relation to one’s self” [73]. According to SCT, employees make a number of comparisons in the work environment. In addition, they use the information gained from these comparisons to make self-evaluations and to determine their attitudes and behaviors [74,75,76]. In this context, SCT can explain the relationships between competitive psychological climate, job envy, organizational justice, and interpersonal citizenship.

## 2. Materials and Methods

### 2.1. Sampling and Data Collection

This study was conducted with a quantitative research method and has a cross-sectional design. The study sample consisted of employees working in four- and five-star accommodation facilities in the Alanya and Manavgat regions of Turkey. In order to reach a larger group of participants, the convenience sampling method, which is a non-probability sampling method, was used in this study. Data collection was carried out between 10 July and 30 September 2023 among employees of 24 different accommodation facilities. The HR managers of the participating companies were contacted, and the objectives of this research study were explained in detail. After interviews with the HR managers of the organizations and providing them with information, the necessary cooperation for the successful completion of this research study was obtained. In the data collection process, the drop and collect method was preferred. In this method, questionnaires were distributed to hotel employees in sealed envelopes and then collected over a period of two weeks. A total of 700 questionnaires were distributed and 582 questionnaires were collected. Four trap questions were included in the questionnaire to measure the level of attention and care that participants gave to the survey questions. These questions aimed to determine how carefully and attentively the respondents read the questions [77]. As a result of the detailed analysis of the trap questions, it was determined that 25 respondents did not answer these questions correctly, and 34 questionnaires were either completely blank or significantly incomplete. In light of these findings, these incomplete or incorrectly completed questionnaires were excluded from the analysis process, thereby improving the quality of the study’s data set, and statistical analyses were conducted on 523 survey data. In terms of gender distribution, 60% of the participants were men (n = 314) and 40% were women (n = 209). Regarding marital status, 61.8% (n = 323) of the participants were single, while 38.2% (n = 200) were married. Regarding age groups, 34.4% (n = 180) of the participants were aged 18–27 years, 45.3% (n = 237) of the participants were aged 28–37 years, 15.5% (n = 81) of the participants were aged 38–47 years, and 4.8% (n = 25) of the participants were aged 48 years and older. This distribution shows that the majority of participants were between the ages of 28 and 37. In terms of educational attainment, the highest proportion of participants had completed high school (53.5%, n = 280), followed by an associate degree (19.9%, n = 104), primary education (14.7%, n = 77), and bachelor’s degree (11.9%, n = 62). This shows that the highest proportion of participants were high school graduates. When analyzing distribution according to tenure, it was found that 19.1% (n = 100) had been working for less than 1 year, 40.5% (n = 212) for 1–3 years, 21.6% (n = 113) for 4–6 years, 10.5% (n = 55) for 7–9 years, and 8.2% (n = 43) for 10 years or more. This distribution indicates that the majority of participants had been with their organization for 1–3 years. Employees from nine different departments participated in the study, including food and beverage (26%), kitchen (12.8%), and housekeeping (15.9%) comprising a total of 54.7% of participants.

### 2.2. Instrument

The questionnaire selected as the measurement tool used in the study consisted of five parts. The first part of the questionnaire included a four-item Competitive Psychological Climate Scale adapted from Fletcher et al. [19] to assess employees’ personal perceptions of the competitive environment. This scale provides an opportunity to assess participants’ perceptions of competition from a broader and more inclusive perspective, rather than specific sales-oriented situations. For example, one of the items states “My manager frequently compares my performance with that of my coworkers” and another states “The amount of recognition you get in this company depends on how you perform compared to others”. All scale items were measured on a five-point Likert scale ranging from 1 (strongly disagree) to 5 (strongly agree). The second part of the questionnaire included the five-item Envy of Others Scale developed by Vecchio [29] to measure employee envy. The scale addresses the emotional states and attitudes that employees feel when compared to other individuals in the workplace. Example items include “It is somewhat annoying to see how others have all the luck in getting the best assignments” and “My supervisor values the efforts of others more than he/she values my efforts”. Responses are rated on a five-point Likert scale (1 = never, 2 = rarely, 3 = seldom, 4 = occasionally, 5 = often). The third part of the questionnaire was designed to assess employees’ perceptions of distributive justice within the organization. This part included the five-item Distributive Justice Scale developed by Niehoff and Moorman [78]. The Distributive Justice Scale measures employees’ perceptions of the extent to which various work outcomes, including pay, work schedule, workload, and job responsibilities, are distributed fairly. Example items are “I consider my workload to be quite fair” and “Overall, the rewards I receive here are quite fair”. The scale is based on a five-point Likert-type rating system (1 = strongly disagree, 5 = strongly agree). High scores on the scale indicate that employees have a strong perception of distributive justice in the work environment. The fourth part of the questionnaire focused on the interpersonal citizenship behavior of employees within the organization. This section included the eight-item task-focused scale developed by Settoon and Mossholder [47]. The items in the scale reveal employees’ tendency to actively help their colleagues and the extent to which they volunteer to do so. Examples of items in this scale are “I take on extra responsibilities in order to help my coworkers when things get demanding at work” and “I help my coworkers with difficult assignments, even when assistance is not directly requested”. High scores on the scale indicate that employees are very helpful and supportive of their coworkers. The scale items are rated on a five-point Likert scale ranging from 1 (strongly disagree) to 5 (strongly agree). The last part of the questionnaire form included questions about the demographic characteristics of the participants, such as age, gender, education, department they work in, and length of service with the organization.

### 2.3. Data Analysis

**Figure 1:**
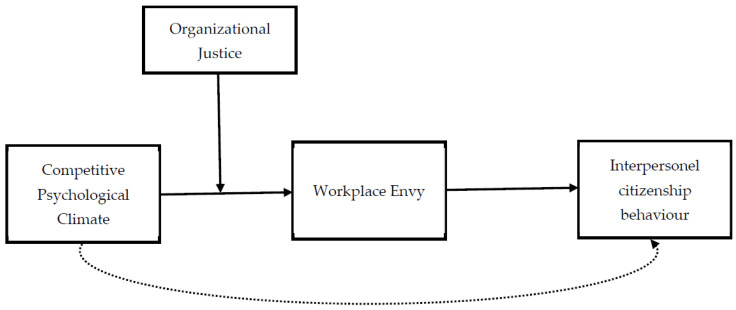
Conceptual model.

This study used the moderated mediation model, which is a statistical model that evaluates moderation and mediation together. The conceptual model of this study is shown in Figure 1. Before proceeding with data analysis, a number of preliminary steps were taken. First, the data set was scanned in detail and checked for missing or erroneous data. As a result of this thorough examination, it was determined that the data set was complete and suitable for analysis. Once the data set was ready for statistical analysis, it was tested for normal distribution. The research model was tested using a two-step approach proposed by Anderson and Gerbing [79]. In the first stage, the accuracy and reliability of the measurement model were tested. In this stage, the convergent and discriminant validity of the scales used in the research were examined. In the second stage, the main hypotheses of the research were analyzed using the PROCESS macro models of Hayes [80]. All these analytical processes were carried out using the SPSS 24.0 (IBM, Armonk, NY, USA) and AMOS 24 (IBM, Armonk, NY, USA) statistical package programs. Covariates were not included in the analysis to avoid complicating the interactions of the main variables examined in the study and to avoid distracting from the main objectives of the study.

## 3. Results

### 3.1. Measurement Model

The CFA results of the measurement model created based on the conceptual structure of this study are shown in Table 1. This measurement model consists of four different conceptual constructs and 22 items representing these constructs. The model fit indices (χ^2^ = 435,209, df = 200, χ^2^/df = 2.18, *p* < 0.001, RMSEA = 0.047, SRMR = 0.03, CFI = 0.98, IFI = 0.98, NFI = 0.96, RFI = 0.95) are consistent with the acceptable values determined by Schermelleh-Engel et al. [81]. These values indicate that the measurement model has an acceptable fit. When the factor loadings in Table 1 were analyzed, it was found that the factor loadings of all items were above 0.50 and statistically significant (*p* < 0.001). In addition, as suggested by Nunnally and Bernstein [82], Cronbach’s alpha values range between 0.93 and 0.97 and these values indicate that the constructs have high internal consistency values. CR and AVE values were examined to assess convergent validity. As stated by Bagozzi and Yi [83], AVE values greater than 0.50 and CR values both greater than 0.70 and greater than AVE values support the convergent validity of the constructs. To test the assumption of normal distribution, skewness and kurtosis values were examined. Since skewness ranged from −0.30 to 1.67 and kurtosis ranged from −0.26 to 2.64, it is evident that the data met the criteria of normal distribution [84].

Table 2 presents the results of the discriminant validity of the conceptual constructs. Discriminant validity determines how well a measure distinguishes a particular concept from other concepts. According to Fornell and Larcker [85], the square root of the AVE of a variable should be greater than the correlations of that concept with other concepts to ensure discriminant validity. Table 2 shows that the square root of the AVE of each concept is greater than its correlations with other concepts. In addition, AVE values that are higher than MSV and ASV values and MaxR(H) values > 0.85 indicate that discriminant validity is ensured. In conclusion, based on the results in Table 1 and Table 2, it was determined that the reliability and validity of the constructs were ensured, and the measurement model was an acceptable model.

This study compared the four-factor model with three alternative models using chi-squared tests. The results in Table 3 show that the four-factor model, which considers each of the four separate constructs as an independent factor, is the best fit for the data. When the four-factor model is compared with the three-, two-, and one-factor models, it was found that the model fit gradually worsened with these alternative configurations. This suggests that the construct is better represented by four distinct factors, and that the fit of the model deteriorates when the constructs are combined. Consequently, the four-factor research model was determined to be the best fit for the data. In addition, the variance inflation factor (VIF) was used to assess multicollinearity in the data set. The results of the study indicate that the variance inflation factor (VIF) for competitive psychological climate (CPC) is 1.25, 1.90 for organizational justice (ORJ), and 2.13 for workplace envy (WPE). All of these VIF values are below the widely accepted conservative threshold of 3 [86], indicating the absence of significant multicollinearity problems within the data set.

### 3.2. Hypothesis Test

This study tested the moderated mediation research model. The moderated mediation effect helps to understand the conditions under which the indirect effects between two variables change. For this analysis, tests were conducted using the PROCESS macro for SPSS developed by Hayes [80]. During the analysis, 95% confidence interval (CI) values were based on 5000 resamples using the bootstrap method. First, the aim was to test whether workplace envy (WPE) has a mediating effect on the relationship between competitive psychological climate (CPC) and interpersonal citizenship behavior (ICB). At this stage, total, direct, and indirect effects on the relationship CPC→WPE→ICP were tested without the moderator variable.

According to the results presented in Table 4, CPC has a positive effect on WPE (β = 0.31, t_(520)_ = 7.98, %95 CI [0.24; 0.39], *p* < 0.001). CPC has a direct negative effect on ICB (β = −0.26, t_(519)_ = −9.93, %95 CI [−0.32; −0.21], *p* < 0.001), and WPE has a negative effect on ICB (β = −0.54, t_(519)_ = −18.78, %95 CI [−0.59; −0.48], *p* < 0.001). The indirect effect of CPC on ICB through WPE is significant (β = −0.17, %95 BCA CI [−0.22; −0.12]). This result indicates that CPC reduces ICB by increasing WPE. In light of these results, we can conclude that CPC has a negative effect on ICB through WPE, and that CPC also has a direct effect on ICB. Thus, CPC has both a direct and a mediated effect on ICB. Accordingly, WPE plays a complementary role by partially mediating the relationship between CPC and ICB. According to these results, hypotheses H1, H2, and H3 are supported.

The results in Table 5 show the moderating effect of organizational justice (ORJ) on the relationship between competitive psychological climate (CPC) and workplace envy (WPE). Accordingly, CPC positively (β = 0.34, t_(519)_ = 13.47, %95 CI [0.29; 0.39], *p* < 0.001), and ORJ negatively (β = −0.52, t_(519)_ = −23.33, %95 CI [−0.56; −0.48], *p* < 0.001) affect WPE. The interaction term (CPC × ORJ) was significant (β = −0.25, t_(519)_ = −11.82, %95 CI [−0.29; −0.21], *p* < 0.001), indicating that organizational justice plays a moderating role in the relationship between competitive psychological climate and workplace envy. The negative value of the interaction term indicates that organizational justice may buffer the negative effect of competitive psychological climate on workplace envy.

**Figure 2:**
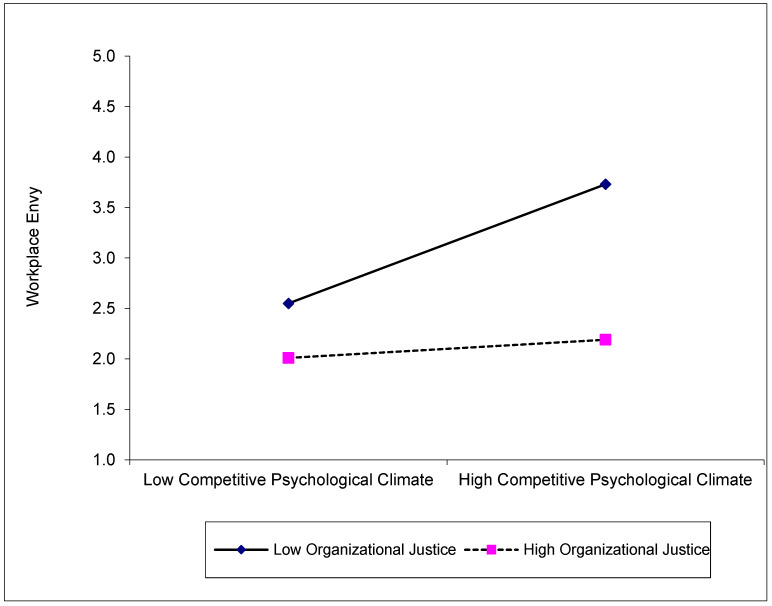
Moderating effect of organizational justice on the relationship between competitive psychological climate and workplace envy.

The results of the conditional effects analysis show that the effect of competitive psychological climate (CPC) on workplace envy (WPE) varies with organizational justice (ORJ). The effects of organizational justice as a moderator variable are shown in Figure 2. In the low ORJ condition, the effect of CPC on WPE is positive and significant (β = 0.65, t_(519)_ = 17.42, %95 CI [0.58; 0.73], *p* < 0.001). However, in the high ORJ condition, CPC loses its effect on WPE (β = 0.03, t_(519)_ = 0.07, %95 CI [−0.07; 0.08]). These results suggest that competitive psychological climate (CPC) is less likely to generate envy (WPE) in employees when perceptions of fairness in organizations are high. In this context, ORJ acts as a mechanism to buffer the potentially negative effects of CPC. This suggests that organizational justice has a significant impact on employees’ perceptions and that perceived justice, as such, can prevent employees from developing negative emotions, even in a competitive environment. These findings suggest that hypothesis H4 is supported.

To test the fifth hypothesis of the study, a moderated mediation regression model was constructed. The results of the moderated mediation regression analysis are shown in Table 6. First, examining the direct relationship between the independent variable competitive psychological climate (CPC) and the dependent variable interpersonal citizenship behavior (ICB), we see that the effect of CPC on ICP is negative, and this effect is statistically significant (β = −0.26, t_(520)_ = −9.93, %95 CI [−0.32; −0.21], *p* < 0.001). Workplace envy (WPE) acting as a mediator also has a negative and statistically significant effect on ICP (β = −0.54, t_(520)_ = −18.78, %95 CI [−0.59; −0.48], *p* < 0.001). This suggests that competitive psychological climate (CPC) and workplace envy (WPE) tend to reduce employees’ citizenship behaviors toward coworkers.

Another important finding of this study is that the indirect effect of CPC on ICP via WPE differs in conditions where ORJ is low or high. Specifically, the indirect effect of CPC on ICP via WPE is more pronounced and significant in conditions with low levels of ORJ (β = −0.35, %95 CI [−0.40; −0.31]). However, in conditions with high levels of ORJ, this indirect effect becomes statistically insignificant (β = −0.02, %95 CI [−0.04; 0.04]). The significant value of the moderated mediation index (β = 0.13, %95 CI [0.11; 0.16]) empirically supports that organizational justice plays a moderating role in the indirect effect of CPC on ICP through WPE. In conclusion, Table 6 clearly shows how the effect of competitive psychological climate on interpersonal citizenship behavior is shaped by both workplace envy and the moderating role of organizational justice. In other words, the results show how competitive psychological climate can affect employees’ interpersonal citizenship behaviors through workplace envy and how this interaction can be moderated by organizational justice. We conclude that the presence of organizational justice can buffer the negative effects of competitive psychological climate on workplace envy as well as its negative indirect effect on interpersonal citizenship behaviors. According to these results, hypothesis H5 is supported.

## 4. Discussion

This study primarily analyzes the effect of competitive psychological climate on workplace envy. The analysis of the data concluded that a competitive work environment has a positive effect on feelings of workplace envy. In most societies, competition, even for personal gain, is generally recognized as a positive phenomenon [87]. In this context, managers in tourism, hospitality, and leisure businesses also find it logical to develop competitive psychological climate to improve employee performance [88]. Competitive climate can even be seen as a factor that can improve factors that are considered positive for many organizations, such as productivity [89], task orientation [19], innovative thinking, individual creative ability [90], career achievement [16], and employee engagement [17]. However, competitive psychological climate, which at first glance seems to be a positive organizational factor, has been found to increase workplace envy. There are understandable reasons for this. The opportunities that can be offered to employees, such as promotion, salary increase, and recognition in the organization, are limited. While a small portion of employees have access to these opportunities, most employees are denied them. In this context, competitive climate causes some employees who do not have access to these limited resources to experience malicious envy [40]. In other words, almost as a natural mechanism, competitive climate gives rise to envy. It is also possible to find studies in the literature that point to an increase in workplace envy due to competitive psychological climate. Eslami and Arshadi [40] conducted a study on the employees of an oil company and found that competitive climate causes envy. In a study conducted by Murtza and Rasheed [71] with the participation of hospitality professionals, it was found that competitive climate causes envy among employees, which leads to a decrease in employee performance. In a study conducted by Mohd. Shamsudin et al., [22] with the participation of bank employees, it was found that in competitive climate, envy increased when employees compared themselves with those whom they considered superior. In a study conducted by Vecchio [91] with the participation of graduate students, it was found that competitive reward systems positively affect individuals’ envy levels. Similarly, in a study conducted by Malone [92] with participants from a variety of backgrounds, particularly graduate students, it was found that competitive psychological climate leads to malicious envy.

As a result of the analyses conducted in this study, it was determined that workplace envy is a factor that reduces interpersonal citizenship behavior. At the same time, workplace envy was found to play a mediating role in the effect of competitive psychological climate on interpersonal citizenship behavior. These findings are consistent with those of previous studies. It is known that competitive psychological climate forces employees to compete for limited resources, and this process causes especially disadvantaged employees to feel envy towards high-performing employees [22,40]. In other words, competitive systems designed to achieve limited rewards create a mechanism that fuels envy in individuals [91]. On the other hand, by its very nature, competition is known to reduce cooperation among employees [29]. In organizations where envy is strongly experienced alongside an increase in internal competition, it is possible that some members of the organization are excluded and some of them attack other individuals in different ways to feel stronger [93]. In these environments, negative relationships between individuals are likely to develop. This is because, under competitive conditions, employees perceive all processes within the organization as a system based on winning or losing [29]. Instead of achieving the formal goals designed by the organization, its processes are treated in the context of “winning” or “losing”, which may pave the way for employee behavior in the competitive process to go beyond ethical principles. These findings indicate that workplace envy is one of the consequences of competitive psychological climate. However, it is also understood that workplace envy is not only an outcome variable, but also one of the causal factors that negatively affect interpersonal relationships. Various existing studies reveal that when employees are envious of what others have, they give up their citizenship behaviors [57]. Eslami and Arshadi [40] stated that workplace envy, which is caused by the opportunities available to a limited number of employees due to competitive climate in the organization, reduces the tendency of some employees to engage in behaviors aimed at benefiting others over time. Li et al. [46] found that envious people lose self-confidence and suffer from an inferiority complex, which reduces their tendency to engage in organizational citizenship behaviors. Cohen-Charash and Mueller [94] even found that envy experienced during this process is likely to trigger the behavior of disrupting others. In this context, individuals who envy high-performing employees may find it difficult to develop positive relationships with others. In an atmosphere of envy with such toxic emotions, relationships between employees are expected to be negatively affected [25]. As a result of the research conducted by Watkins [54] on university employees and students, it was concluded that envy negatively affects interpersonal citizenship behavior. Kim et al. [44] conducted a study with the participation of front-line hotel employees and found that there is a negative relationship between envy and interpersonal citizenship behavior. Thompson et al. [95] conducted a study with the participation of employees and found that as workplace envy increases, citizenship behavior indirectly decreases. Ghadi [57] determined that envious employees who wish ill to their coworkers avoid exhibiting citizenship behaviors. In addition to these findings, and from a different perspective, Shu and Lazatkhan [96], as a result of a study on corporate employees, determined that social undermining behavior also increases as envy in the organization increases. In another study conducted by Cohen-Charash and Mueller [94] on individuals taking courses at a university, it was found that there was a positive relationship between an increase in envy levels and interpersonal counterproductive work behaviors. Our findings, in line with the results of previous studies, suggest that envy is a consequence of competitive psychological climate, and that envy is a cause that reduces interpersonal citizenship behaviors.

Another important finding of this study is that the effect of competitive psychological climate on workplace envy varies depending on employees’ perceptions of distributive justice. In other words, the negative effect of competitive climate on workplace envy is significantly moderated by the moderating role of distributive justice perceptions. At the same time, the mediating role of workplace envy in the relationship between competitive psychological climate and interpersonal citizenship behavior (ICB) was found to be significantly different according to employees’ distributive justice perceptions. The results of the study show that when employees perceive distributive justice, the mediating effect of workplace envy on the relationship between competitive psychological climate and interpersonal citizenship behaviors weakens. This suggests that a fair work environment may alleviate the negative emotional states that may be caused by competition and increase employees’ tendency to help each other.

Previous studies in the literature provide insights that help make sense of this finding. Competitive psychological climates are known to negatively affect employee relationships, cause envy, reduce cooperation, and even lead to counterproductive workplace behaviors [19,21]. However, when managers provide justice within the organization, opportunities for cooperation among employees can develop even under highly competitive conditions. In this context, organizational justice can become a factor that reduces some of the negative effects of competitive psychological climate [97]. In other words, when employees are treated fairly within the organization, they believe that they will face fair outcomes even under conditions of intense competition [98]. Employees’ belief that they will face fair outcomes may prevent the development of some negative behaviors. This is because under conditions of fairness, no matter how intense the competition, employees know that they face lower risks for themselves [72]. In other words, creating transparent reward systems and ensuring organizational justice can mitigate the negative effects of competitive climate [99]. The evaluations presented in previous studies indicate that organizational justice is a factor that reduces the negative effects of competitive psychological climate. On the other hand, previous studies also provide notable evaluations of the relationship between organizational justice and envy. Ben-Ze’ev [100] stated that the feeling of envy is often accompanied by perceived injustice and even said that “envy seems to include the desire to eliminate inequality” (p.551). Cohen-Charash and Mueller [94] found that organizational injustice also triggers envy and even the development of counterproductive work behaviors. Similarly, Malone [92] pointed out that a lack of justice in an organization is one of the factors that causes envy. In this context, it is understood that organizational justice is a factor that reduces the negativity arising from competitive psychological climate; thus, organizational justice plays a role in buffering the effect of competitive psychological climate on workplace envy. As a result, this research study illuminates the intricate relationships between competitive psychological climate, workplace envy, and perceived distributive justice. Our findings underscore the importance for organizations of creating a healthy competitive climate among employees while maintaining a fair work environment and minimizing negative emotional states such as envy. Achieving this balance will not only help employees optimize their individual performance but will also strengthen organizational commitment and collaborative culture.

Our research explores the multifaceted nature of competitive psychological climate within organizational contexts. While traditionally lauded as a catalyst for organizational efficiency [19,89], our study reveals a more complex and nuanced dimension. Although competition can foster individual achievements [16,90], it can also create an environment that promotes workplace envy. This is particularly evident when opportunities for advancement, recognition, and rewards are perceived as limited and unfairly distributed. Such an environment not only fosters malicious envy but also undermines citizenship behavior, as our findings confirm. Our results show that workplace envy can reduce the positive effects of competitive climates on interpersonal citizenship behavior. This highlights the potential for envy to cause behaviors that harm interpersonal relationships and hinder workplace cooperation. This finding highlights that envy can lead to disruptive behaviors, which can strain interpersonal relationships and cooperation within the workplace. These outcomes are particularly detrimental in sectors such as hospitality, where teamwork and interpersonal harmony are essential.

Our study highlights the ameliorative impact of distributive justice. We found that when employees perceive fairness in the allocation of resources and rewards, the negative effects of competitive climate on workplace envy and, consequently, on interpersonal citizenship behavior are significantly reduced. Cultivating a fair and equitable environment is crucial in mitigating the negative effects of competition. Organizational justice is essential in achieving a balance between competition and cooperation. Therefore, we recommend a more thoughtful approach to promoting competitive environments within organizations. To achieve a balance that nurtures healthy competition, while simultaneously upholding distributive justice and fostering a cooperative ethos, management strategies should focus on enhancing transparency in reward systems and ensuring equitable distribution of resources. To achieve a balance that nurtures healthy competition, while simultaneously upholding distributive justice and fostering a cooperative ethos, management strategies should focus on enhancing transparency in reward systems and ensuring an equitable distribution of resources. On the other hand, employees’ concerns about the losses they will face if they fail in competitive processes can be reduced, which matters because the great sense of loss experienced as a result of failure may lead to unethical behaviors among employees. Different measures can be taken specifically to prevent workplace envy. Individuals with a high level of emotional maturity can be preferred when selecting job candidates. Participative management policies can be preferred and employees can be involved in decision-making processes. More opportunities for cooperation between employees can be created. High-achieving staff can be considered as mentors [31]. Social activities can be organized to improve social communication between employees. Thus, the development of friendly relationships can be supported.

We believe that our research findings make a theoretical contribution to SCT. This is because SCT states that employees make various comparisons in the work environment and shape their attitudes and behaviors as a result of these comparisons [74,75,76]. It is known that the concepts of competitive psychological climate, organizational justice, and workplace envy, which are the focus of this research, are all based to some extent on comparison behavior. While comparisons with less qualified individuals allow for a positive emotional state, it is known that comparisons with more highly qualified individuals pave the way for negative emotions [101]. Considering that individuals generally compare themselves with more highly qualified people [102], the fact that the resulting negative emotions affect individuals’ attitudes and behaviors at work is seen as a situation that can be explained within the framework of SCT. In this regard, competitive psychological climate, which directly affects workplace envy and indirectly affects interpersonal citizenship behaviors, is considered in this study to be explained in the context of SCT.

## 5. Conclusions

In this study, the relationships between competitive psychological climate, job envy, interpersonal citizenship behavior, and perceived organizational justice were examined within the framework of data obtained from employees of accommodation companies. In this context, one of the most striking findings of this study is related to the effects of competitive psychological climate. Although competitive psychological climate is considered a factor that would increase the performance of employees as well as the productivity of the organization, it is understood that competitive climate that is not effectively managed can turn into a damaging factor rather than bring the expected benefits. Accordingly, it is once again understood that it would not be correct to categorically accept the factor of interemployee competition in organizations as a directly positive or directly negative factor [19]. Thus, “management must maintain a balance between competition and cooperation in order to obtain the best performance from the available human resources” (p.1410) [71]. In other words, the results of this study revealed that competitive psychological climate that is not effectively managed would increase workplace envy and negatively affect interpersonal relationship behavior. Therefore, preventing competitive psychological climate from reaching a level that triggers workplace envy should be considered a critical line in terms of employee performance and productivity. On the other hand, another notable finding of this study is that the effect of competitive psychological climate on workplace envy can be regulated by organizational justice. This finding shows that organizational justice is one of the key factors in the proper management of competitive psychological climate. Accordingly, when managers notice the possibility of workplace envy becoming a harmful factor, they should propose approaches to promote cooperation by reducing competition within the organization [25] and focus on solutions that will eliminate problems in organizational justice. Otherwise, the competitive psychological climate that develops in an inequitable environment should be seen as an expected outcome that will fuel workplace envy and consequently damage teamwork and cooperation, which are very important for hospitality businesses [103]; that is, interpersonal citizenship behaviors will decrease.

## 6. Limitations and Future Research

The results of this research study should be considered in the context of the limitations of the research. As in any research study, there are some limitations in this study. First, due to technical conditions, a cross-sectional method was preferred in the study, but we believe that the use of longitudinal methods by future researchers will contribute to more comprehensive evaluations. Second, convenience sampling was preferred to non-probability sampling in this study due to difficulties in data collection. Although convenience sampling is a quick, easy, and inexpensive sampling method, it may be necessary to be more cautious in generalizing the results obtained with this method. Therefore, we believe that it is important for future researchers to prefer probability sampling methods. Finally, this study was designed using quantitative methods. However, the use of qualitative methods in addition to quantitative methods in future research efforts will help to obtain more comprehensive information on the subject.

## Figures and Tables

**Table 1:** Result of measurement model.

Construct	Items	Factor Loadings	S.E.	t Values	CR	AVE	Cronbach’s Alpha
Competitive Psychological Climate	CPC1	0.875		Fixed	0.91	0.72	0.91
	CPC2	0.787	0.044	18.624 ***			
	CPC3	0.831	0.039	24.976 ***			
	CPC4	0.924	0.032	28.911 ***			
Workplace Envy	WPE1	0.861		Fixed	0.93	0.73	0.93
	WPE2	0.797	0.046	22.648 ***			
	WPE3	0.856	0.042	26.078 ***			
	WPE4	0.845	0.040	25.317 ***			
	WPE5	0.916	0.036	29.467 ***			
Organizational Justice	ORJ1	0.918		Fixed	0.94	0.76	0.94
	ORJ2	0.702	0.035	19.731 ***			
	ORJ3	0.863	0.031	30.499 ***			
	ORJ4	0.864	0.028	30.120 ***			
	ORJ5	0.948	0.024	38.261 ***			
Interpersonal Citizenship Behavior	ICB1	0.884		Fixed	0.94	0.65	0.94
	ICB2	0.723	0.035	20.059 ***			
	ICB3	0.792	0.037	23.740 ***			
	ICB4	0.781	0.036	22.836 ***			
	ICB5	0.780	0.037	22.926 ***			
	ICB6	0.820	0.035	25.159 ***			
	ICB7	0.804	0.034	24.060 ***			
	ICB8	0.867	0.032	28.110 ***			

*** *p* < 0.001.

**Table 2:** Results of discriminant validity.

	MSV	ASV	MaxR(H)	CPC	WPE	ORJ	ICB
CPC	0.25	0.12	0.93	[0.85]			
WPE	0.47	0.34	0.94	0.35 ***	[0.85]		
ORJ	0.41	0.21	0.95	0.03	−0.64 ***	[0.87]	
ICB	0.47	0.31	0.94	−0.50 ***	−0.69 ***	0.48 ***	[0.81]

ASV = average shared variance, CPC: competitive psychological climate, ICB: interpersonal citizenship behavior, ORJ: organizational justice, MSV = maximum shared variance, WPE: workplace envy. Values in square brackets [ ] are the square root values of AVE. *** *p* < 0.001.

**Table 3:** Comparison of alternative measurement models for main constructs.

Models	X²	df	X²/df	CFI	RMSEA	Model Comparison		
						∆X²	∆df	*p* (∆X²)
1. Hypothesized four-factor model ^a^	435.210	200	2.18	0.98	0.047	-	-	-
2. Three-factor model ^b^	1911.570	206	9.28	0.83	1.126	1476.36	6	1476.36
3. Two-factor model ^c^	2458.150	208	16.63	0.68	1.713	2022.94	8	2022.94
4. One-factor model ^d^	4550.690	209	21.77	0.52	0.199	4115.48	9	4115.48

^a^ = competitive psychological climate; organizational justice; workplace envy; interpersonal citizenship behavior. ^b^ = competitive psychological climate; organizational justice + workplace envy; interpersonal citizenship behavior. ^c^ = competitive psychological climate + organizational justice + workplace envy; interpersonal citizenship behavior. ^d^ = competitive psychological climate + organizational justice + workplace envy + interpersonal citizenship behavior.

**Table 4:** Results of mediation analysis.

	Mediator (WPE)					Dependent (ICP)				
Antecedent	β	SE	t Statistic	LLCI	ULCI	β	SE	t Statistic	LLCI	ULCI
CPC	0.31	0.04	7.98	0.24	0.39	−0.26	0.03	−9.93 ***	−0.32	−0.21
WPE	-	-	-	-	-	−0.54	0.03	−18.78 ***	−0.59	−0.48
	R^2^ = 0.11 F_(1,521)_ = 63.68, *p* < 0.001					R^2^ = 0.55 F_(2,520)_ = 320.40, *p* < 0.001				
Total effect of CPC → ICP						−0.43	0.03	−13.12 ***	−0.50	−0.37
Direct effect CPC → ICP						−0.26	0.03	−9.83 ***	−0.32	−0.21
Bootstrap Indirect Effects CPC → WPE → ICP						−0.17	0.02	-	−0.22	−0.12

CPC: competitive psychological climate, ICB: interpersonal citizenship behavior, WPE: workplace envy. *** *p* < 0.001.

**Table 5:** Result of moderating effect of organizational justice.

	Dependent (WPE)				
Antecedent	β	SE	t Statistic	LLCI	ULCI
CPC	0.34	0.03	13.47	0.29	0.39
ORJ	−0.52	0.02	−23.33	−0.56	−0.48
CPC × ORJ	−0.25	0.02	−11.82	−0.29	−0.21
	R^2^ = 0.63 F_(3,519)_ = 295.58, *p* < 0.001				
Conditional effects of ORJ	β	SE	t statistic	LLCI	ULCI
Low ORJ: CPC → WPE	0.65	0.04	17.42 ***	0.58	0.73
High ORJ: CPC → WPE	0.03	0.04	0.07	−0.07	0.08

*** *p* < 0.001.

**Table 6:** Results of moderated mediation model.

Antecedent	β	SE	t Statistic	LLCI	ULCI
CPC → ICP	−0.26	0.03	−9.93 ***	−0.32	−0.21
WPE → ICP	−0.54	0.03	−18.78 ***	−0.59	−0.48
	R^2^ = 0.55 F_(2,520)_ = 320.40, *p* < 0.001				
Conditional indirect effects		β	SE	LLCI	ULCI
CPC → WPE × ORJ_(Low)_ → ICP		−0.35	0.02	−0.40	−0.31
CPC → WPE × ORJ_(High)_ → ICP		−0.02	−0.02	−0.04	0.04
Index of moderated mediation		0.13	0.01	0.11	0.16

CPC: competitive psychological climate, ICB: interpersonal citizenship behavior, ORJ: organizational justice, WPE: workplace envy. *** *p* < 0.001.

## Data Availability

The data that support the findings of this study are available from the corresponding author upon reasonable request.
